# Conjugation-based genetic manipulation of *Fusobacterium animalis*

**DOI:** 10.1128/mbio.01714-25

**Published:** 2025-07-30

**Authors:** Duhyun Ko, Wendy S. Garrett

**Affiliations:** 1 Department of Immunology & Infectious Diseases, Harvard T. H. Chan School of Public Health 1857 , Boston, Massachusetts, USA; 2 Harvard T. H. Chan Microbiome in Public Health Center, Boston, Massachusetts, USA; 3 Infectious Disease and Microbiome Program, Broad Institute of MIT and Harvard, Cambridge, Massachusetts, USA; 4 Department of Molecular Metabolism, Harvard T. H. Chan School of Public Health 1857 , Boston, Massachusetts, USA; 5 Department of Medical Oncology, Dana-Farber Cancer Institute and Harvard Medical School, Boston, Massachusetts, USA; Corporación CorpoGen, Bogotá, Colombia

**Keywords:** *Fusobacterium animalis*, conjugation, genetic manipulation, hydrogen sulfide, *megL*

## Abstract

*Fusobacterium* is an oncobacterium associated with colorectal cancer (CRC) development. Despite that *F. animalis* (*Fa*) dominates the CRC niche in humans, studies on the pathogenesis of *Fusobacterium* have been limited by the paucity of genetically tractable *Fa* strains. Here, we constructed a conjugative DNA transfer system for genetic manipulation of *Fa* clinical isolates. A mobilizable shuttle plasmid can be transferred by conjugation from *Escherichia coli* to various *Fa* strains, including *Fa* 7_1, which has been considered genetically intractable using electroporation methods. The conjugation efficiency of *Fa* 7_1 was further improved by ethanol treatment. Next, we manipulated the *E. coli* donor strain to express *Fusobacterium* methyltransferases, which can protect plasmids from degradation by restriction systems in *Fa* recipient strains. With this donor strain, we could isolate transconjugants of *Fa* harboring chromosomally integrated plasmid. As a test case, we disrupted *megL* which is involved in cysteine metabolism and hydrogen sulfide (H_2_S) production. The plasmid integrated into the *megL* locus was stably maintained without antibiotic selective pressure. The *megL* disruption in *Fa* 7_1 significantly decreased the level of H_2_S production *in vitro* in the presence of cysteine and resulted in lower cecal H_2_S level in a mouse colonization model. The same conjugation-based system was successfully applied to another *Fa* strain CTI-1, although this strain showed 10-fold lower conjugation efficiency compared to *Fa* 7_1. Altogether, this work suggests that conjugation is an efficient method for genetic manipulation of *Fa* strains, allowing us to mechanistically study their pathogenicity in CRC at the molecular level.

**IMPORTANCE:**

Among the *Fusobacterium* species associated with colorectal cancer (CRC), *F. animalis* (*Fa*) is the most clinically relevant species showing distinct genetic features. However, due to its genetic intractability, little is known experimentally about the molecular factors of *Fa* contributing to CRC development. Here, we showed that foreign DNA can be transferred from *Escherichia coli* to *Fa* via conjugation. The conjugation system was developed by expressing *Fusobacterium*-derived methyltransferases in the *E. coli* donor strain to transfer methylated plasmid to *Fa*. Finally, the *megL* gene, encoding an enzyme responsible for hydrogen sulfide (H_2_S) production, was successfully disrupted in *Fa* strains. The effects of the *megL* disruption in *Fa* on H_2_S production were verified both *in vitro* and *in vivo*. This conjugation-based approach would be applied to not only *Fa* but also a broader range of *Fusobacterium* species, expanding our understanding of their virulence traits.

## INTRODUCTION

*Fusobacterium* is a gram-negative anaerobic bacterium found in the human oral cavity and gastrointestinal tract. *Fusobacterium* is associated with colorectal cancer (CRC), and its abundance is increased in human colonic adenomas and CRC tumors compared to normal tissues (1–3). *Fusobacterium* adheres to CRC cells, activates oncogenic responses, and impairs antitumor immunity, but only a few virulence factors have been experimentally interrogated to date, Fap2, FadA, and RadD (4–7). This limitation is due to the lack of robust genetic tools for *Fusobacterium*. Transformation of *Fusobacterium* has often relied upon electroporation methods to which most fusobacterial strains are recalcitrant. As *F. nucleatum* ATCC 23726 (*Fn* ATCC 23726) exhibits relatively high electroporation efficiency, this strain has been mostly used as a model organism to study the roles of *Fusobacterium* in CRC development. However, a recent study found that *F. animalis* (*Fa*), rather than *Fn*, is a major CRC-associated *Fusobacterium*, supporting the importance of focusing on *Fa* as a primary target for mechanistic studies (8). Although several genetic tools based on electroporation have been proposed to increase the transformation efficiency of *Fusobacterium* (9–11), manipulation of *Fa* is still challenging. Sonoporation was used for DNA transfer but applied to only one strain of *F. polymorphum* to date (12, 13). Thus, an alternative approach for *Fa* genetic manipulation is strongly warranted.

Conjugation is a bacterial mechanism for horizontal gene transfer through physical contact between donor and recipient cells. For conjugative DNA transfer, plasmids should carry an origin of transfer (*oriT*) recognized by a relaxase protein (14). The relaxase binds to the *oriT* region and generates a single-stranded DNA (ssDNA) by cleaving specific sites, and then, the relaxase-ssDNA complex is transferred from donor cells to recipient cells (14). Although a conjugation method was recently used to manipulate *F. necrophorum* and *F. polymorphum* (15, 16), it has not been applied to *Fa*. In this study, we constructed the conjugation system for *Fusobacterium* and successfully applied it for genetic manipulation of CRC-relevant *Fa* strains, which are known to be genetically intractable by electroporation.

## RESULTS

### Construction of a conjugation system for *Fusobacterium*

**Fig 1:**
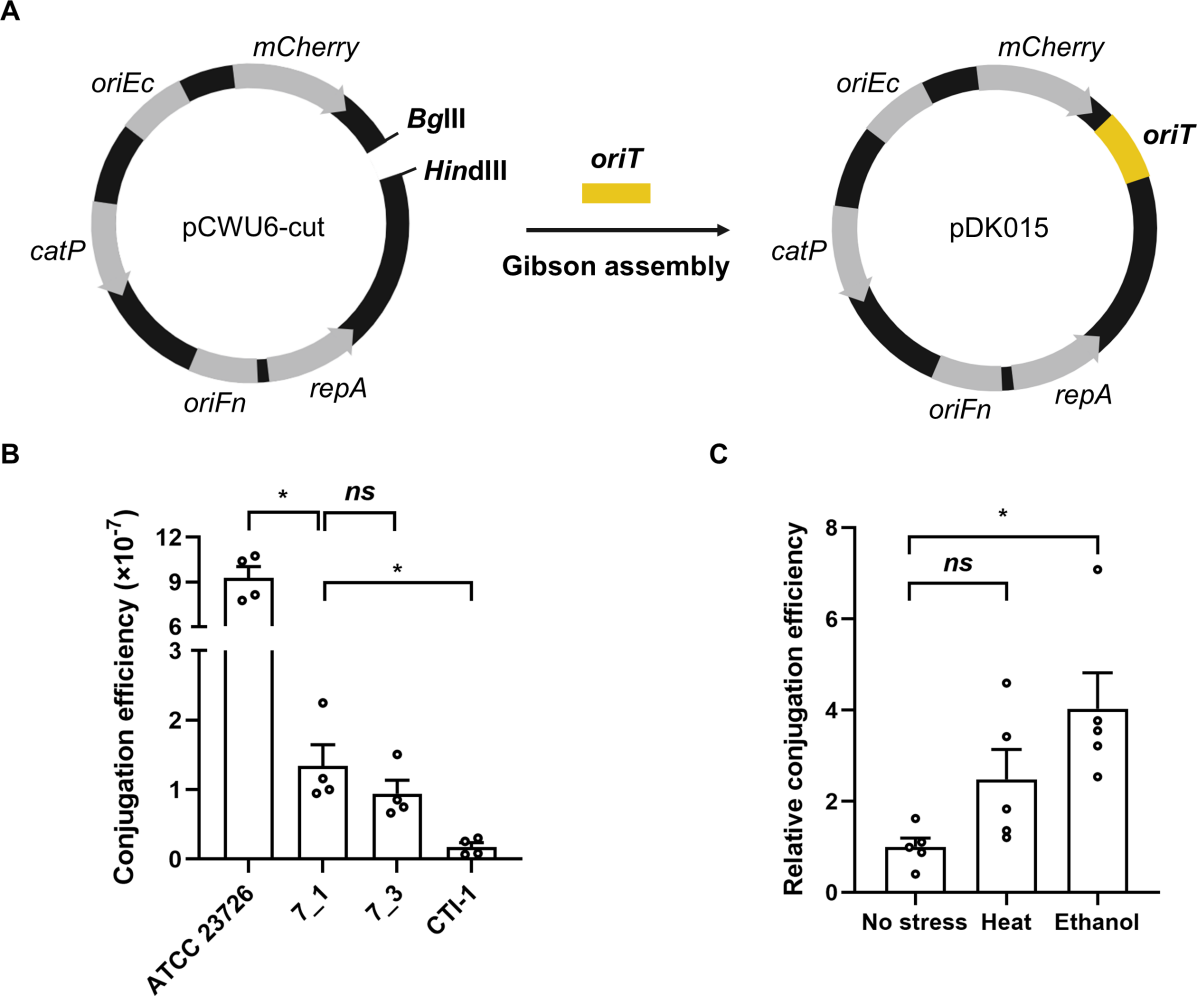
Construction of conjugation system for *Fusobacterium*. (**A**) Construction of a mobilizable *E. coli-Fusobacterium* shuttle vector pDK015. The *Bg*lII-*Hin*dIII-digested pCWU6 (pCWU6-cut) was assembled with *oriT* fragment from pDN19. (**B**) Conjugation efficiency of *Fn* ATCC 23726 and *Fa* strains (7_1, 7_3, and CTI-1) was calculated as the ratio of the number of transconjugant cells to the number of recipient cells. (**C**) Effect of stress treatment on conjugation efficiency of *Fa* 7_1. Relative conjugation efficiency of *Fa* 7_1 without stress treatment was set as 1. Error bars represent the standard error of the mean (SEM). Statistical significance was determined by the Mann-Whitney *U*-test. *, *P* < 0.05; *ns*, not significant.

To test whether *Fusobacterium* can acquire foreign DNA via conjugation, a mobilizable *Escherichia coli-Fusobacterium* shuttle vector pDK015 was constructed by cloning the *oriT* fragment into pCWU6 (Fig. 1A). pCWU6 is a well-established shuttle vector conferring thiamphenicol resistance to *Fusobacterium* (17)*. E. coli* MFD*pir* was used as a donor strain. This strain is an auxotroph for diaminopimelic acid (DAP), which enables simple removal of the donor cells from donor-recipient mixed cultures after conjugation. When conjugation was conducted using *Fn* ATCC 23726 as a recipient, we were able to obtain approximately 10^−6^ transconjugants per recipient cell, suggesting the applicability of a conjugation method to other *Fusobacterium* strains (Fig. 1B).

Next, we examined the conjugation efficiency of three different *Fa* strains 7_1, 7_3, and CTI-1. *Fa* 7_1, isolated from inflamed human colon tissue, has been shown to potentiate CRC in mouse models, and two *Fa* strains 7_3 and CTI-1 were isolated from CRC tumors (3, 18, 19). The pDK015 vector was successfully transferred to all *Fa* strains with an efficiency of 10^−7^ to 10^−8^ transconjugants per recipient cell (Fig. 1B). As an exposure of certain bacteria to environmental stresses such as heat and ethanol increases their conjugation efficiency (20–23), we tested whether these stresses could enhance conjugative DNA transfer to *Fa*. Conjugation efficiency of *Fa* 7_1 was not significantly affected by the heat treatment but increased fourfold by the ethanol treatment (Fig. 1C). These findings were remarkable as *Fa* 7_1 has been considered a genetically intractable strain (10). Although the conjugation efficiency of the tested *Fa* strains was variable, these results indicated that genetic materials can be transferred to *Fa* through conjugation.

### Construction of conjugation-based gene knock-out system targeting *megL*

Conventional gene knock-out systems require homologous recombination. Given a low rate of homologous recombination in *Fusobacterium* (11), we decided to generate the isogenic mutant by one-step single-crossover recombination. For this, a mobilizable backbone vector pDK016 was constructed by cloning *oriT* into pHS31 (https://doi.org/10.6084/m9.figshare.29318471). The thiamphenicol resistance conferred by the pDK016-based vector would result from chromosomal integration because the vector is unable to replicate independently in *Fusobacterium* due to the absence of the origin of replication for *Fusobacterium* (*oriFn*).

**Fig 2:**
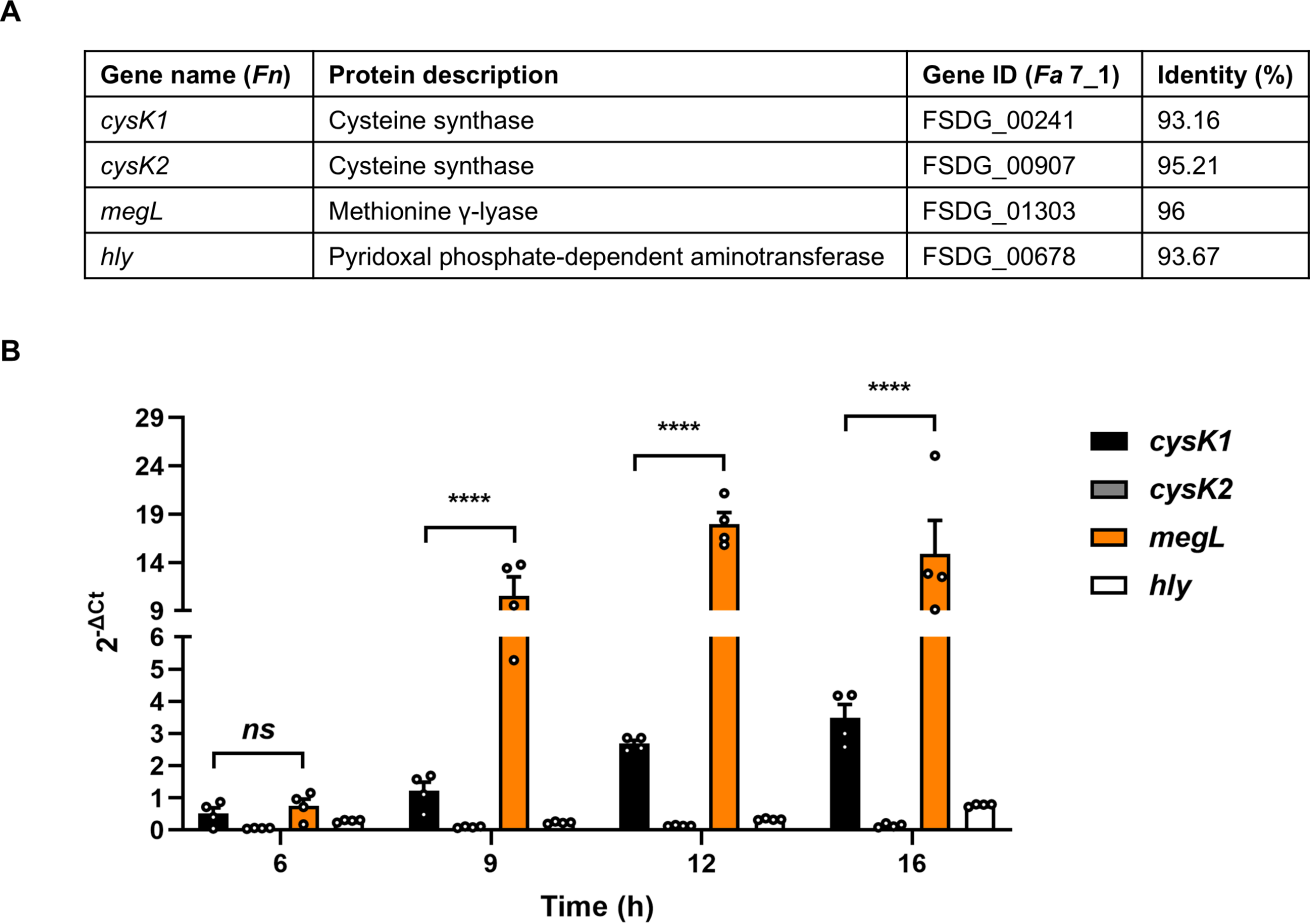
Expression of genes associated with cysteine metabolism in *Fa* 7_1. (**A**) Genes of *Fa* 7_1 homologous to *cysK1*, *cysK2*, *megL*, and *hly* of *Fn* ATCC 23726 were searched by BLAST. Percent identity was calculated based on the amino acid sequence of each protein. (**B**) Total RNAs were isolated from *Fa* 7_1 at each indicated timepoint. The expression of *cysK1*, *cysK2*, *megL*, and *hly* was determined by RT-qPCR. Error bars represent the SEM. Statistical significance was determined by two-way analysis of variance (ANOVA) with Tukey’s post hoc test. ****, *P* < 0.0001; *ns*, not significant.

Hydrogen sulfide (H_2_S) facilitates the proliferation, angiogenesis, and metastasis of CRC cells and suppresses the antitumor effects of chemotherapy as well as immunotherapy (24–26). H_2_S can be generated during cysteine metabolism not only by mammalian tissues but also by the human microbiota, including *Fusobacterium*. In *Fn*, four enzymes FN1220 (CysK1), FN1055 (CysK2), FN1419 (MegL), and FN0625 (Hly) have been identified to produce H_2_S from cysteine (27–31). One study revealed that colonic tumorigenesis by *Fn* is exacerbated when mice are given additional cysteine, suggesting the pro-cancer effects of the bacterium-produced H_2_S (32). However, the exact mechanism by which the microbial metabolite H_2_S facilitates CRC progression is unknown. Thus, we sought to generate a mutant strain deficient in H_2_S production in the *Fa* 7_1 genetic background as a test of our method. By BLAST analysis, we found the genes of *Fa* 7_1 homologous to *cysK1*, *cysK2*, *megL*, and *hly* of *Fn* (Fig. 2A). To predict the gene responsible for H_2_S production in *Fa* 7_1, the expression of the four genes was compared at different time points. Except for the 6 h time point, the *megL* expression was highest among the four genes across all time points (Fig. 2B). The expression of *cysK2* and *hly* was negligible, and although the *cysK1* expression slightly increased at the 16 h time point, the level was much less than that of *megL* (Fig. 2B). These results led us to select the *megL* gene as a target gene that may mainly contribute to H_2_S production in *Fa* 7_1.

**Fig 3:**
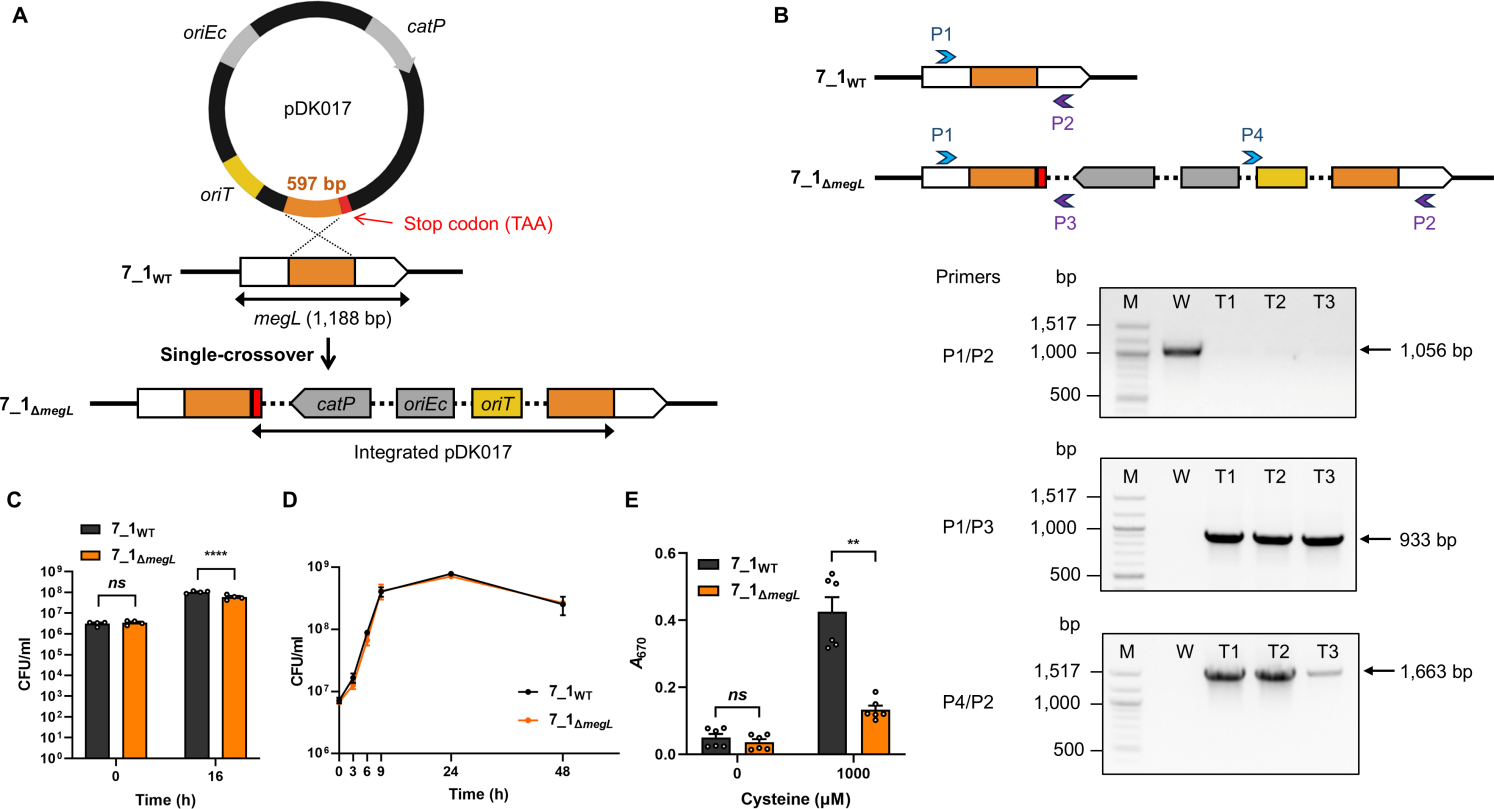
Targeted disruption of the *megL* gene in the *Fa* 7_1 genetic background. (**A**) Schematic representation of the integration of pDK017 into the *megL* locus through single-crossover recombination. (**B**) Confirmation of the chromosomal integration of pDK017 in the *Fa* 7_1 genetic background using the pairs of primers P1/P2, P1/P3, and P4/P2. Blue and purple arrows indicate forward and reverse primers, respectively. Lane M, molecular weight marker; lane W, PCR products from wild-type *Fa* 7_1; lanes T1, T2, and T3; PCR products from three isolated transconjugants. (**C**) Growth yield of the *Fa* strains in minimal medium was determined at the 16 h time point. (**D**) Growth kinetics of the *Fa* strains in rich medium was monitored at each indicated time point. (**E**) H_2_S production (*A*_670_) of the *Fa* strains with or without cysteine was measured by methylene blue assay. Error bars represent the SEM. Statistical significance was determined by the Mann-Whitney *U*-test. **, *P* < 0.005; ****, *P* < 0.0001; *ns*, not significant. 7_1_WT_, wild type; 7_1_Δ*megL*_, *megL* mutant.

To generate an integration vector pDK017 targeting *megL*, a 597 bp of *megL* internal fragment was cloned into pDK016, and a stop codon was added to downstream of the fragment to prevent the translation of unexpected protein (Fig. 3A and https://doi.org/10.6084/m9.figshare.29318471). Conjugation was conducted using *E. coli* MFD*pir* and *Fa* 7_1 as donor and recipient strains, respectively, but we could not obtain any transconjugants. The result indicated that the conjugation efficiency should be improved to overcome the poor homologous recombination of *Fusobacterium*.

One of the hurdles in the genetic manipulation of *Fusobacterium* is its diverse restriction-modification systems that consist of restriction endonucleases and methyltransferases. The endonucleases recognize methylation patterns of specific DNA sequences and digest differentially modified foreign DNA. It was previously reported that methylation of plasmids using *Fn*-derived methyltransferases greatly increases electroporation efficiency (10). To apply this strategy to our conjugation system, we generated a new *E. coli* donor strain that expresses the *Fn* methyltransferases and transfers the modified plasmids to the recipient strain. For this, the *mrr* gene encoding restriction endonuclease Mrr was first deleted in *E. coli* MFD*pir* to allow the replication of DNA exhibiting *Fusobacterium*-methylation patterns. By using the new donor strain, we isolated three transconjugants after conjugative transfer of pDK017 to *Fa* 7_1. The PCR results showed that pDK017 is exactly integrated into the *megL* locus in *Fa* 7_1 (Fig. 3B). The chromosomal integration was also confirmed by DNA sequencing.

### Stability of the chromosomal integration of pDK017

As the *megL* gene was disrupted by single-crossover recombination, two identical copies of the *megL* internal fragment are harbored in the chromosome of *megL* mutant (Fig. 3A). Thus, it is possible that homologous recombination spontaneously occurs in the *megL* mutant, leading to loss of the integrated pDK017 and reverting to wild type. To test this hypothesis, we passaged the *megL* mutant 10 times in the absence of thiamphenicol, randomly selected 50 colonies at each passage, and examined their antibiotic resistance. All colonies during 10 passages were resistant to thiamphenicol, indicating that the chromosomal integration of pDK017 is stably maintained even without antibiotic selective pressure.

### *megL* plays a key role in H_2_S production of *Fa* 7_1

Before evaluating the level of H_2_S production in wild type and *megL* mutant, we tested if the *megL* gene affects the growth of *Fa*. In the minimal medium, the growth yield of *Fa* 7_1 was slightly decreased by the *megL* disruption, suggesting that MegL-related metabolic pathways are important for optimal growth of *Fa* (Fig. 3C). In contrast, the *megL* mutant showed a similar growth rate to wild type in rich medium (Fig. 3D). Thus, H_2_S production of the *Fa* strains was measured in rich medium by the methylene blue assay. Without the addition of cysteine, both wild type and *megL* mutant produced H_2_S at a minimal level (Fig. 3E). However, the addition of cysteine to wild type dramatically increased the H_2_S level, indicating that cysteine is a main source for H_2_S production in *Fa* 7_1 (Fig. 3E). The *megL* mutant also produced H_2_S from cysteine, but the level was much lower than that of wild type (Fig. 3E). These results showed that the *megL* gene is required for full production of H_2_S in *Fa* 7_1.

**Fig 4:**
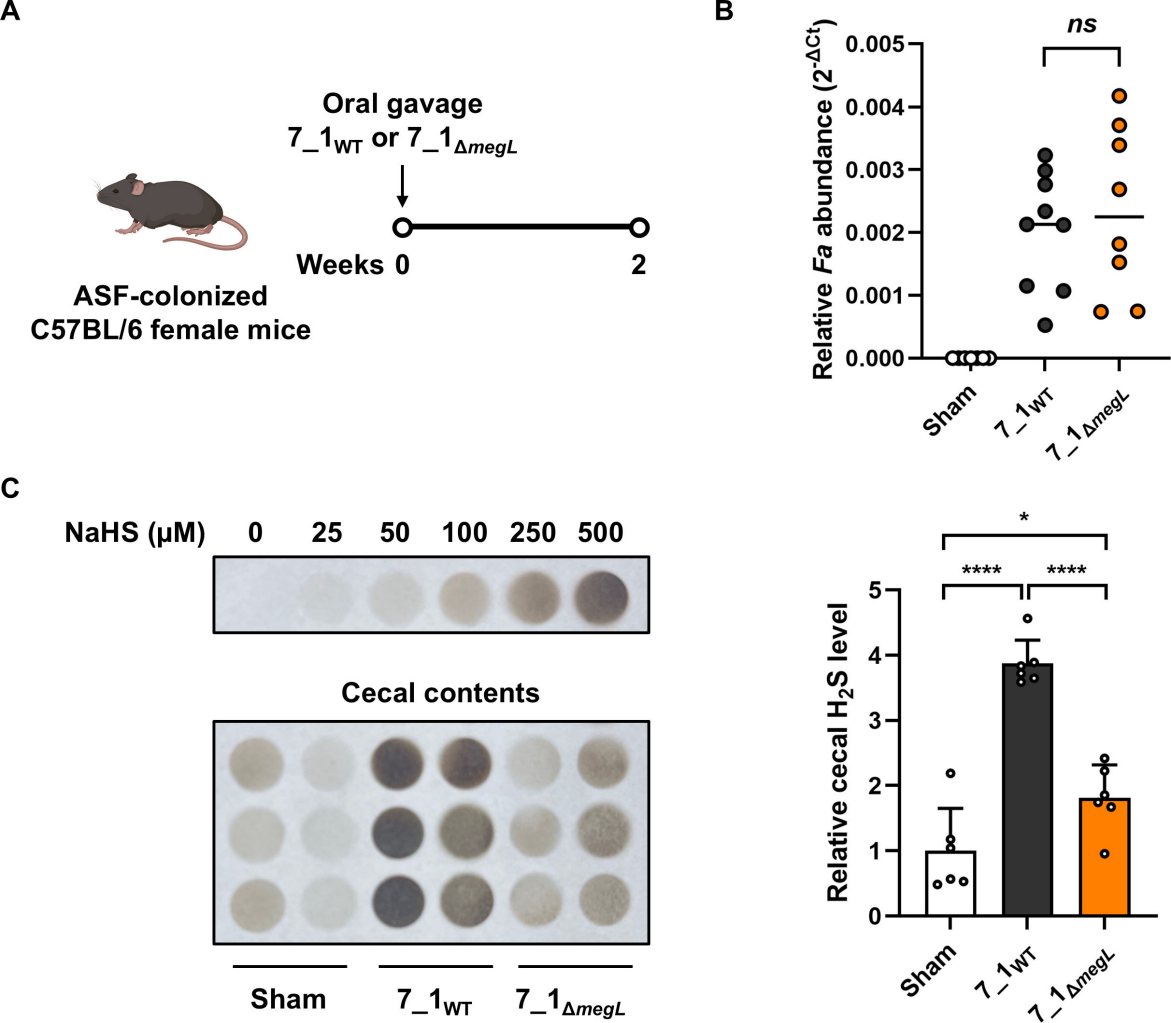
Colonization and H_2_S production of *Fa* in the ASF mouse model. (**A**) Eight-week-old ASF female mice were orally gavaged with 7_1_WT_ or 7_1_Δ*megL*_ and sacrificed after 2 weeks. (**B**) Relative *Fa* abundance (2^−ΔCt^) in the feces from sham or *Fa*-gavaged mice. (**C**) H_2_S level in the cecal contents from sham or *Fa*-gavaged mice was measured by lead acetate assay. A representative image of lead acetate paper after reaction with NaHS and the cecal contents is shown. The cecal H_2_S level was quantified by densitometry analysis and calculated using a standard curve generated with the indicated concentrations of NaHS. Relative cecal H_2_S level in sham mice was set as 1. Error bars represent the SEM. Statistical significance was determined by one-way ANOVA with Tukey’s post hoc test. *, *P* < 0.05; ****, *P* < 0.0001; *ns*, not significant. 7_1_WT_, wild type; 7_1_Δ*megL*_, *megL* mutant.

To determine the effects of the *megL* disruption in *Fa* on H_2_S production *in vivo*, we used mice colonized with the altered Schaedler’s flora (ASF), a simplified microbial community of 8 bacterial strains (33). As *Fa* 7_1 colonizes the ASF mice by a single oral gavage (34), the mice were orally gavaged once with the wild type or *megL* mutant *Fa* (Fig. 4A). The fecal abundance of *Fa* in the mice gavaged with the *megL* mutant was comparable to that observed in the mice gavaged with wild type, indicating that the *megL* mutant has no defect in colonization (Fig. 4B). Then, the H_2_S level in the cecal contents from sham or *Fa*-colonized mice was measured by the lead acetate assay. Cecal H_2_S level was markedly elevated in the wild-type-colonized mice compared to the sham mice (Fig. 4C). Colonization of the *megL* mutant slightly increased the cecal H_2_S level, but the extent of this increase was significantly lower than that induced by colonization of wild type (Fig. 4C). These results suggested that the *megL* disruption in *Fa* 7_1 is functionally maintained *in vivo* without affecting its colonization capacity.

### Application of the conjugation-based genetic manipulation system to *Fa* CTI-1

**Fig 5:**
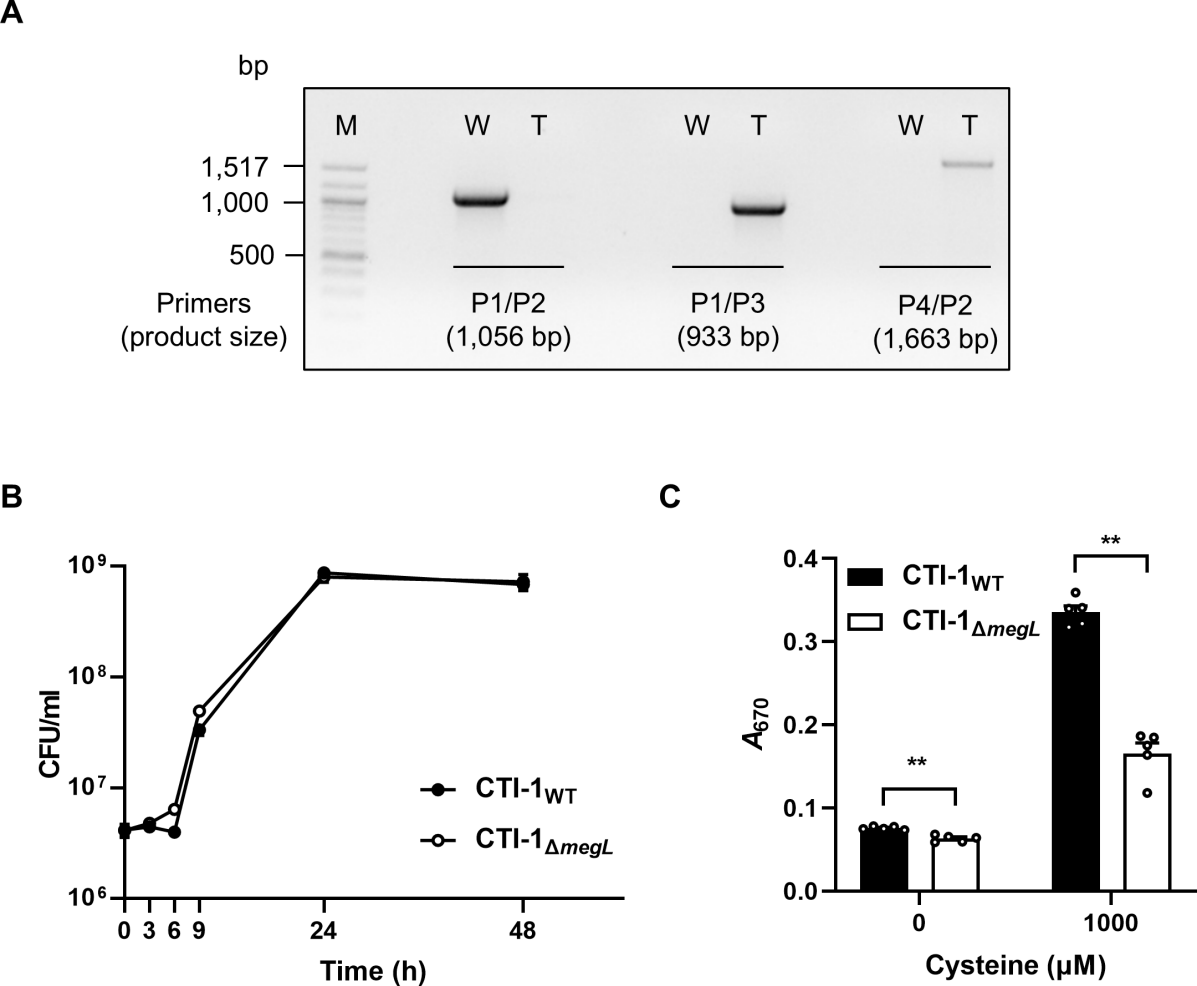
Application of the conjugation-based genetic manipulation system to *Fa* CTI-1. (**A**) Confirmation of the chromosomal integration of pDK017 in the *Fa* CTI-1 genetic background using the pairs of primers P1/P2, P1/P3, and P4/P2 (indicated in Fig. 3B). Lane M, molecular weight marker; lane W, PCR products from wild-type *Fa* CTI-1; lane T, PCR products from the isolated transconjugant. (**B**) Growth kinetics of the *Fa* strains was monitored at each indicated time point. (**C**) H_2_S production (*A*_670_) of the *Fa* strains with or without cysteine was measured by the methylene blue assay. Error bars represent the SEM. Statistical significance was determined by the Mann-Whitney *U*-test. **, *P* < 0.005. CTI-1_WT_, wild type; CTI-1_Δ*megL*_, *megL* mutant.

To determine whether this conjugation-based system could be applicable to other *Fa* strains, pDK017 was transferred to the *Fa* CTI-1 strain, whose conjugation efficiency is 10-fold lower than that of *Fa* 7_1 (Fig. 1B). After selection by thiamphenicol resistance, we obtained the *megL* mutant in the *Fa* CTI-1 genetic background (Fig. 5A). Similar to *Fa* 7_1, the chromosomal integration of pDK017 had no effect on the growth of *Fa* CTI-1 and resulted in lower H_2_S production (Fig. 5B and C). These results suggested that the conjugation-based genetic manipulation system could be extensively applied to various *Fa* strains.

## DISCUSSION

It has been well-studied in preclinical models that *Fa* 7_1 is highly invasive for CRC cells, modulates tumor-immune microenvironment, and promotes colonic tumorigenesis (3, 19, 34, 35). However, the molecular factors underpinning *Fa* 7_1’s tumorigenic potential remain relatively under-explored due to its genetic intractability. Among the *Fa* strains, only *Fa* 21_1A has been transformed by electroporation so far (36), but its virulence has not been established yet. In this study, we showed that the *Fa* strains, including *Fa* 7_1, can be transformed by a conjugation method. Conjugation efficiency was further increased when the *Fa* 7_1 cells were exposed to ethanol before mating, which may be attributed to an alteration in membrane permeability for DNA transfer (37, 38). The potential effects of other stresses, such as detergents and pH change, on the conjugation efficiency of *Fusobacterium* should be investigated in the future.

Restriction-modification systems are defense systems used by bacteria to distinguish modified self-DNA from unmodified nonself-DNA. Restriction endonucleases recognize and degrade the nonself double-stranded DNA, acting as a barrier to DNA transfer. Conjugation may have an advantage over electroporation because DNA is transferred to recipient cells as a single strand. Nevertheless, bacterial restriction-modification systems are a key factor in determining the efficiency of conjugative DNA transfer. For example, loss of restriction activity in the recipient strain results in a 6.5-fold increase in conjugation efficiency (39). Expression of recipient-derived modification systems in the donor strain also increases DNA transfer frequency (40). Similarly, we developed the conjugation system by manipulating the donor strain to express two different *Fusobacterium*-derived methyltransferases. This development led us to isolate transconjugants of *Fa* 7_1 and *Fa* CTI-1 harboring chromosomally integrated plasmids, overcoming the low recombination rate of *Fusobacterium*. Together with the modulation of DNA methylation patterns, decreasing the number of methylation sites in plasmids could be another strategy to avoid the restriction activity in the recipient strain (41). We are currently working on this to further improve the conjugation efficiency and introduce diverse genetic tools for a broader range of *Fusobacterium* species.

H_2_S is a gasotransmitter endogenously produced in various tissues, mediating multiple biological processes such as gene transcription and translation, cellular bioenergetics, and cell cycle regulation (42). Colon tumor tissues produce higher amounts of H_2_S compared to adjacent normal mucosa tissues (24). H_2_S can stimulate tumor growth and angiogenesis and suppress the effects of immunotherapy by altering the tumor microenvironment (24, 25). The gut microbiota are also major producers of H_2_S from inorganic sulfate and organic sulfur compounds such as cysteine, methionine, and taurine (43). Among the sulfur-metabolizing microbiota, cysteine-degrading bacteria have been reported to be more abundant in human guts than sulfate-reducing bacteria (44). Interestingly, microbial genes related to cysteine/methionine metabolism such as *megL* are more prevalent in CRC patients than healthy individuals (43). We constructed the *megL* mutant in both *Fa* 7_1 and *Fa* CTI-1 genetic backgrounds and verified the function of MegL by measuring H_2_S production from cysteine. Importantly, we confirmed that the cecal H_2_S level is lower in mice colonized with the *megL* mutant compared to the wild-type-colonized mice. Mechanistic studies using the *megL* mutant may provide insights into the effects of *Fa*-derived metabolite H_2_S on CRC development.

The *megL* gene was successfully disrupted by the chromosomal integration of the plasmid, but there are some limitations in our method. For example, although we confirmed the stability of the integrated plasmid under the lack of antibiotic selective pressure, it is still possible that genomic rearrangements occur and lead to unintended mutations in *Fa*. In addition, if the target gene is part of an operon, integration of the plasmid could induce polar effects, and another selection marker is required for its complementation. The main disadvantage is that this method cannot be applied to disruption of multiple genes. These limitations would be overcome through the generation of double-crossover recombinants in *Fa*, following an improvement in the conjugation efficiency. In summary, we constructed a conjugation-based genetic manipulation system for *Fusobacterium* and applied it to the *Fa* strains for *megL* disruption. This study would facilitate the development of genetic tools for many *Fusobacterium* species that have been difficult to genetically manipulate by electroporation, allowing us to understand their roles in tumorigenesis at the molecular level.

## MATERIALS AND METHODS

### Bacterial strains, plasmids, and growth conditions

The strains and plasmids used in this study are listed in Table 1. *Fusobacterium* strains were grown at 37°C on fastidious anaerobe agar (Neogen, Lansing, MI) supplemented with 5% defibrinated sheep blood (FAA) or in tryptic soy broth supplemented with hemin (5 µg/mL) and menadione (1 µg/mL) (sTSB) anaerobically using a vinyl chamber (Coy Lab Products, Grass Lake, MI). M9 minimal medium supplemented with 1% (wt/vol) peptone, 0.5 mM MgSO_4_, 0.1 mM CaCl_2_, and 0.05% (wt/vol) cysteine was used to determine the growth yield of the *Fa* strains. *E. coli* strains were grown at 37°C in Luria-Bertani (LB) medium aerobically. When required, antibiotics were added to the medium at the following concentrations: ampicillin, 100 µg/mL; chloramphenicol, 20 µg/mL; thiamphenicol, 5 µg/mL; and tetracycline, 10 µg/mL. Bacterial growth was monitored by counting colony-forming units (CFUs).

**TABLE 1:** Bacterial strains and plasmids used in this study

Strain or plasmid	Description^*a*^	Reference or source
Bacterial strains		
*F. nucleatum* ATCC 23726	Wild type	American Type Culture Collection
*F. animalis*		
7_1	Wild type	(19)
7_3	Wild type	(18)
CTI-1	Wild type	
7_1 Δ*megL*	7_1 *megL*::pDK017; Tm^R^	This study
CTI-1 Δ*megL*	CTI-1 *megL*::pDK017; Tm^R^	This study
*E. coli*		
MFD*pir*	Wild type; donor strain; auxotroph for DAP	(45)
MFD*pir* Δ*mrr*	MFD*pir* with Δ*mrr*	This study
Plasmids		
pCWU6	*E. coli-Fusobacterium* shuttle vector; Cm^R^ Tm^R^	(17)
pDN19	Mobilizable vector with *oriT*; Tc^R^	(46)
pDK015	pCWU6 with *oriT* (the *Bgl*II-*Hin*dIII-digested pCWU6 was assembled with *oriT* from pDN19); Cm^R^ Tm^R^	This study
pKD46	Expression vector for arabinose-inducible lambda Red recombination system; Ap^R^	(47)
pKD3	Template for amplification of Cm resistance cassette; Ap^R^ Cm^R^	(47)
pCP20	Expression vector for FLP recombinase; Ap^R^ Cm^R^	(48)
pDJSVT26	Expression vector for *Fn*-methyltransferases; Ap^R^	(10)
pHS31	Gene-inactivation vector; Cm^R^ Tm^R^	(49)
pDK016	pHS31 with *oriT* (the *Eco*RI-*Sac*I-digested pHS31 was assembled with *oriT* from pDN19); Cm^R^ Tm^R^	This study
pDK017	pDK016 with *megL*-stop (the *Bam*HI-*Nhe*I-digested pDK016 was assembled with *megL* internal fragment with stop codon [TAA]); Cm^R^ Tm^R^	This study

^*a*^ Tm^R^, thiamphenicol resistance; Cm^R^, chloramphenicol resistance; Tc^R^, tetracycline resistance; Ap^R^, ampicillin resistance.

### Plasmid construction

For the construction of a mobilizable vector pDK015 and pDK016, the *oriT* region was amplified from pDN19, and the fragments were assembled with BglII-HindIII-digested pCWU6 and EcoRI-SacI-digested pHS31, respectively. For the construction of pDK017, the *megL* internal region in *Fa* 7_1 was amplified using primers MEGL_F and MEGL_R and assembled with BamHI-NheI-digested pDK016 (https://doi.org/10.6084/m9.figshare.29318471). The primer MEGL_R was designed to carry an in-frame stop codon (TAA).

### Conjugation

*E. coli* donor strains grown for 9 h and *Fusobacterium* recipient strains grown for 6 h were harvested by centrifugation at 3,500 × *g* for 5 min. The cells were washed with their growth medium and mixed in 1 mL of sTSB. After centrifugation at 1,500 × *g* for 5 min, the mixed cells were spotted on Columbia agar supplemented with hemin (5 µg/mL), menadione (1 µg/mL), and DAP (300 µM) and mated for 18–20 h anaerobically. When indicated, the *Fa* 7_1 cells were exposed to heat (42°C) or 12% (vol/vol) ethanol for 15 min and washed before mixing with the *E. coli* donor cells. The *E. coli* donor cells were counterselected by the DAP auxotrophy, and the transconjugants of *Fusobacterium* were selected by thiamphenicol resistance.

### Manipulation of *E. coli* donor strain

*E. coli* MFD*pir* Δ*mrr* was generated by the standard lambda Red recombination method (47). Briefly, *E. coli* MFD*pir* was first transformed with the pKD46 vector expressing Red recombinase under the arabinose-inducible promoter. Then, the chloramphenicol resistance cassette was amplified from pKD3 using primers MRR-F and MRR-R, designed to carry 5′ and 3′ flanking regions of the *mrr* gene (https://doi.org/10.6084/m9.figshare.29318471). The amplified fragment was introduced to *E. coli* MFD*pir* carrying pDK46, replacing the *mrr* gene with the chloramphenicol resistance cassette. The cassette was removed by pCP20, generating *E. coli* MFD*pir* Δ*mrr*. Then, pDJSVT26 expressing two methyltransferases of *Fn* ATCC 23726 under the constitutive Anderson promoter (10) was introduced to *E. coli* MFD*pir* Δ*mrr*. The *E. coli* MFD*pir* Δ*mrr* carrying pDJSVT26 was used as a donor strain for conjugative transfer of pDK017 to *Fa* strains.

### RNA purification and quantitative RT-qPCR analysis

The *Fa* 7_1 cells were lysed in QIAzol Lysis Reagent (Qiagen, Germantown, MD) after pretreatment with Max Bacterial Enhancement Reagent (Thermo Fisher Scientific, Waltham, MA). Total bacterial RNAs were isolated using the Direct-zol RNA Miniprep Kit (Zymo Research, Irvine, CA). DNase was additionally treated using the TURBO DNA-free Kit (Thermo Fisher Scientific). Preparation of cDNA and RT-qPCR was performed as described previously (50) using the appropriate pairs of primers (https://doi.org/10.6084/m9.figshare.29318471). Relative expression of each gene was calculated by 2^−ΔC*t*^ normalized to the expression of *pgk* and *pgi* as the internal references (51).

### Methylene blue assay

The *Fa* strains grown in the sTSB medium for 16 h were incubated for 1 h with or without cysteine. The cultures were centrifuged at 4,000 × *g* for 5 min, and 100 µL of supernatant was mixed with 900 µL of 0.1 M potassium phosphate buffer (pH 8). Then, 100 µL of 43 mM N,N-dimethyl-p-phenylenediamine sulfate in 7.2 M HCl and 100 µL of 148 mM FeCl_3_ in 1.2 M HCl were added sequentially. The *A*_670_ was measured after incubation at 37°C for 20 min.

### Animal experiments

C57BL/6 female mice colonized with the ASF were housed in semi-rigid gnotobiotic isolators and transferred to individually ventilated isolator cages for Fa gavage. Eight-week-old ASF colonized mice were orally gavaged once with the Fa strains (~10^9^ CFU in 100 μL) and maintained for an additional 2 weeks.

### Fecal DNA extraction and qPCR analysis

Fecal DNA was extracted by the phenol-chloroform method as described previously (52). The same amount of fecal DNA was used for qPCR with the appropriate pairs of primers targeting eubacterial (53) and *Fa* 16S rRNA gene, respectively (https://doi.org/10.6084/m9.figshare.29318471). The relative abundance of *Fa* in the feces was calculated by 2^−ΔC*t*^, where ΔC*t* = (C*t* value of *Fa* 16S rRNA gene) – (C*t* value of eubacterial 16S rRNA gene). All qPCR was performed on a Stratagene MX3005P (Agilent Technologies, Santa Clara, CA) using the KAPA SYBR FAST Universal kit (Kapa Biosystems, Cape Town, South Africa).

### Lead acetate assay

The lead acetate assay was performed as described previously (52). Briefly, the cecal contents from sham or *Fa*-gavaged mice were collected into pre-weighed 1.5 mL tubes. For homogenization, cold-PBS supplemented with 10 mM cysteine and 1 mM pyridoxal-5-phosphate was added at a ratio of 200 µL per 100 mg of cecal contents. The 96-well plate containing 200 µL of homogenate was covered with lead acetate paper tightly and incubated at 37°C for 24 h. The cecal H_2_S level was quantified by densitometry analysis using ImageJ software and calculated using a standard curve generated with different concentrations of NaHS ranging from 0 to 500 µM.

### Statistical analysis

Statistical analyzes were performed as described in the figure legends using GraphPad Prism 9.0 (GraphPad Software, San Diego, CA).
